# Cognitive Reserve Measurement: A Mobile Application for Detecting, Monitoring and Enhancing Cognitive Resilience and Reducing Alzheimer's Risk

**DOI:** 10.1002/alz70858_107765

**Published:** 2025-12-26

**Authors:** Arshia A Khan, Patricia Khashayar

**Affiliations:** ^1^ University of Minnesota Duluth, Duluth, MN, USA; ^2^ University of Minnesota, Minneapolis, MN, USA

## Abstract

**Background:**

Cognitive Reserve (CR) is the brain's inherent capacity to withstand neurological changes and maintain cognitive functionality despite pathological or age related changes. It also provides valuable insight into one's resilience to Alzheimer's disease pathology. Epidemiological research indicates, attainment of higher cognitive reserve reduces the risk of developing Alzheimer's disease by maintaining better brain functionality.

**Methods:**

Here, we propose a novel mobile application, Cognitive Reserve Measurement, designed for comprehensive assessment, longitudinal monitoring and targeted augmentation of CR to alleviate the risk of Alzheimer's disease. To meticulously track behaviours germane to cognitive well‐being, our application employs interfaces like a home screen, journal data, and game screen to encourage user engagement and regular participation in cognition‐boosting activities. The established Cognitive Reserve Index Questionnaire (CRIq) is seamlessly integrated to provide a robust quantitative evaluation.

**Results:**

App was developed using Flutter and Dart for cross‐platform functionality, the application's backend relies on Firebase for secure and real‐time storage of user data, such as journal entries and CRI scores. Ensuring data security is a top priority given the sensitive nature of cognitive health. Upon initial setup, user's attain their baseline based score on Cognitive Reserve Index Questionnaire (CRIq) score. The Journal section prompts users daily to reflect on activities from the previous day and the Journal Data section is for tracking activities that influence cognitive health shall purposefully track user habits stimulus for CR (e.g. regular physical activity, social engagement, sleep regimen, stress management etc.) and progress over time through sophisticated data visualization. Users can also engage in brain training games designed to enhance memory and reasoning skills. Currently the app is in the testing phase with a prototype being evaluated for usability and engagement to promote cognitive resilience and support healthy aging.

**Conclusion:**

Aiming to address the escalating global need for effective cognitive health intervention within an increasing geriatric population, the application leverages the accessibility of mobile technology and provides a tool for precise measurement and optimization of CR. The foundational rationale for this application underscores the pivotal role of CR in preserving healthy brain functionality across lifespan, eventually preventing or delaying Alzheimer's disease onset.

